# EMDS-7-FSCIL: a benchmark for Few-Shot Class-Incremental Learning in environmental microorganism recognition

**DOI:** 10.3389/fmicb.2026.1770528

**Published:** 2026-02-10

**Authors:** Jinyi Zhou, Yinuo Zhang, Sihang Xu, Yangfan Hu, Jinhui Zeng, Yimin Yin

**Affiliations:** 1 School of Intelligent Manufacturing, Hunan First Normal University, Changsha, China; 2 Rocket Force University of Engineering, Xi'an, China; 3 School of Computer Science and Mathematics, Fujian University of Technology, Fuzhou, China; 4 College of Meteorology and Oceanography, National University of Defense Technology, Changsha, China; 5 School of Mathematics and Statistics, Hunan First Normal University, Changsha, China

**Keywords:** class-incremental learning, deep learning, EMDS-7 dataset, environmental microorganism, Few-Shot Learning

## Abstract

Deep learning–based environmental microorganism recognition in a dynamic world demands models capable of recognizing novel classes in new tasks. However, it is hindered by data scarcity, high annotation costs, and the plasticity–stability dilemma. Few-Shot Class-Incremental Learning (FSCIL) aims to address these challenges, yet a dedicated benchmark for environmental microorganism recognition remains absent. To bridge this gap, we establish the first FSCIL benchmark for environmental microorganism recognition and propose a unified evaluation protocol on the EMDS-7 dataset. We systematically reproduce 10 representative FSCIL methods: CEC, FACT, SAVC, PFR, ADBS, Comp, TEEN, Limit, BiDist, and CLOSER, and conduct comprehensive comparative experiments under a consistent implementation setting. We report multidimensional evaluation metrics, including per-session accuracy, average accuracy across sessions, and performance drop rate to quantify long-term performance degradation, along with thorough performance analyses. Our results reveal that SAVC and FACT achieve the highest overall accuracy, while PFR demonstrates more stable performance at the cost of a lower accuracy ceiling. In contrast, CLOSER and BiDist exhibit substantially weaker performance. Overall, our benchmark reveals that FSCIL methods effective on generic image benchmarks do not directly transfer to environmental microorganism recognition tasks, necessitating task-specific adaptations. This work provides a reproducible foundational platform to enable fair comparisons and accelerate future research on FSCIL for environmental microorganism recognition.

## 1 Introduction

**Figure 1:**
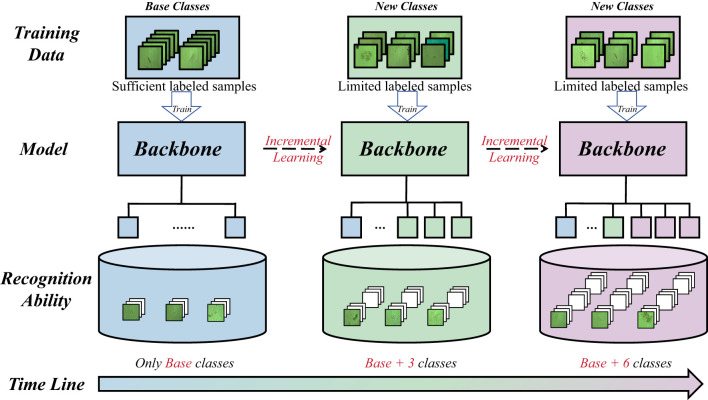
Illustration of the FSCIL-based environmental microorganism recognition process. As time progresses, microbial communities in real-world monitoring scenarios continuously evolve and new microorganism classes emerge. Given the high cost of data collection, and annotation, the model should continuously learn novel classes from only a few labeled examples while maintaining performance on previously learned classes.

Environmental microorganisms are vital components of ecosystems, contributing to nutrient cycling, water purification, and overall ecological stability (Yin et al., 2024). At the same time, they serve as sensitive bioindicators for assessing water quality, detecting pollution sources, and monitoring ecological balance (Pluym et al., 2024). Accurate recognition of these microorganisms is therefore essential for understanding environmental dynamics and guiding sustainable management practices. However, traditional recognition methods–such as manual microscopic examination, biochemical assays, and molecular sequencing–are time-consuming, labor-intensive, and costly (Arbefeville et al., 2024), and they often require substantial expert involvement. Recent advances in computer vision and deep learning have enabled Automated Microorganism Detection and Recognition (AMDR), allowing models to analyze morphological and textural features in microscopic images. Representative work also includes task-specific architectures (e.g., LCU-Net; Zhang J. et al., 2021) designed for environmental microorganism microscopy segmentation. These approaches significantly improve recognition efficiency and objectivity. In real-world environmental microbial image classification and monitoring, deep learning models are typically static and can only recognize categories they were trained on. However, monitoring tasks are diverse, and environments change continuously, so new categories that are not included in the training set will inevitably appear in the field, and these new categories often have very few samples and are difficult to annotate. For example, in long-term online monitoring of environmental microbes (illustrated in Figure 1), as time evolves, the model needs to learn categories it has not encountered before. Ideally, a model should be able to rapidly adapt to new categories under sparse-sample conditions while not forgetting previously learned ones. Few-Shot Class-Incremental Learning (FSCIL) is well-suited to this requirement.

These challenges align closely with the goals of FSCIL, which aims to extend a trained model to additional, previously unseen categories using only a few labeled examples, while retaining knowledge of previously learned classes (Zhang et al., 2025). The central technical difficulty, from an FSCIL perspective, is balancing stability and plasticity: mitigating catastrophic forgetting of old classes while remaining adaptable to new data (De Lange et al., 2021). This challenge is particularly relevant to environmental microorganism monitoring. Deep models for these tasks are typically trained in a static, closed-set manner and, as a result, can only reliably operate on the categories observed during training. In practice, monitoring tasks are diverse and may involve categories that are absent from the original training set, for which labeled samples are often scarce. Therefore, an ideal system would incorporate such categories under sparse supervision without degrading performance on previously learned ones. Despite this need, progress has been hindered by the lack of standardized benchmarks that reflect real-world ecological imaging complexity and support incremental, realistic evaluation protocols (Panaïotis et al., 2025).

To address this limitation, we adopt the Environmental Microorganism Dataset Seventh Version (EMDS-7) (Yang et al., 2023) as the foundation for constructing the first benchmark dedicated to FSCIL in environmental microorganism recognition. EMDS-7 is a publicly available microscopic dataset designed for object detection and ecological analysis of microorganisms. It contains 2,365 images across 41 microorganism categories and 13,216 annotated objects collected from natural lakes and rivers in Shenyang, China. The dataset exhibits notable ecological authenticity, capturing realistic conditions such as overlapping microorganisms, uneven staining, illumination variations, and background impurities. Compared with earlier EMDS versions, EMDS-7 expands taxonomic diversity and provides richer annotations, making it well-suited for studying both class-incremental and few-shot learning scenarios.

Building on EMDS-7, this work constructs an FSCIL evaluation protocol that partitions the dataset into base and incremental sessions to emulate real-world monitoring conditions in which new classes emerge under severe annotation scarcity. Under this framework, we evaluate representative FSCIL baselines, including rehearsal-based iCaRL (Rebuffi et al., 2017) and graph-adaptive classifier evolution CEC (Zhang C. et al., 2021). We further include forward-compatible training via FACT (Zhou D. et al., 2022) and contrastive regularization via SAVC (Song et al., 2023), and compare against a recent decision-boundary adaptation strategy via ADBS (Li et al., 2025). The benchmark reveals several intrinsic challenges of EMDS-7, such as significant intra-class variation and small inter-class differences, which complicate the stability-plasticity trade-off during continual adaptation.

**Figure 2:**
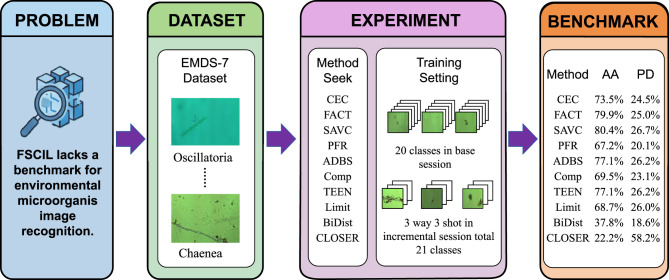
Overall workflow for constructing the EMDS-7 FSCIL benchmark. This figure presents the experimental setup and benchmark results of few-shot class-incremental learning on the EMDS-7 dataset. The base session contains 20 classes, followed by incremental sessions under a 3-way 3-shot setting; the incremental classes contain 21 classes. Multiple representative methods are compared in terms of average accuracy and performance degradation, providing a comprehensive evaluation of their classification capability and stability in environmental microorganism FSCIL scenarios.

In summary, this study introduces the first benchmark for FSCIL in environmental microorganism recognition, leveraging EMDS-7 as a realistic and challenging evaluation platform. By bridging environmental image analysis and adaptive deep learning, the benchmark provides a standardized foundation for testing FSCIL algorithms under ecologically plausible, data-scarce conditions. Figure 2 summarizes the end-to-end workflow of our benchmark construction and evaluation.

## 2 Related work

### 2.1 Few-Shot Learning

Few-Shot Learning (FSL) aims to enable models to recognize novel categories using only a few labeled samples per class (Tsoumplekas et al., 2024). This paradigm is particularly important in scientific domains such as environmental monitoring and biological taxonomy, where data collection is expensive and sample acquisition is constrained (Bennequin et al., 2022; Lim et al., 2025).

Existing FSL methods mainly follow three approaches. First, data augmentation-based methods expand limited datasets by generating or transforming samples (e.g., via semantic priors or adversarial generation). Second, meta-learning-based methods (e.g., MAML-style learning-to-learn) optimize for rapid task adaptation across episodes (Hospedales et al., 2021). Finally, graph-based methods explicitly model relationships between support and query samples for relational reasoning (Zhou X. et al., 2022).

Although these methods have achieved success on standard benchmarks such as Omniglot and miniImageNet, they are mostly evaluated on static, clean, and well-separated tasks, which can deviate from realistic fine-grained and evolving deployments (Bennequin et al., 2022). This differs substantially from real-world scenarios (e.g., environmental microbiology) where new morphotypes continually emerge, and the learner must adapt under extreme sample scarcity over time; consequently, conventional FSL alone is often insufficient for continuously evolving environments (Lim et al., 2025).

### 2.2 Class-Incremental Learning

Class-Incremental Learning (CIL) enables machine learning models, most commonly neural network, to recognize an expanding set of classes introduced progressively over time. Unlike FSL, CIL typically assumes that each newly introduced class is supported by a relatively sufficient number of training samples, while requiring the model to preserve discriminative performance on previously learned classes as new ones are acquired. The central challenge of CIL is mitigating catastrophic forgetting, a phenomenon in which the model's recognition performance on earlier classes degrades significantly as it learns new ones.

Existing CIL approaches can be broadly categorized into several methodological paradigms. Exemplar-based replay methods (e.g., iCaRL; Rebuffi et al., 2017 ) retain a small set of historical samples and leverage knowledge distillation to maintain decision boundaries for previously learned classes. Constraint or regularization-based methods (e.g., LUCIR; Hou et al., 2019 ) restrict updates to network parameters or feature representations in order to enforce representational consistency across incremental learning stages. Representation-level distillation methods perform feature-space distillation to ensure the stability of the embedding space throughout the incremental learning process. Although these methods have demonstrated strong performance on large-scale natural image benchmarks, they generally rely on the availability of sufficient, clean, and well-curated labeled data at each incremental stage and assume well-curated visual domains. Such assumptions limit their direct applicability to scientific domains such as environmental microbiome analysis, where samples from novel classes are often extremely scarce and are further characterized by substantial noise and high intra-class variability.

### 2.3 Few-Shot Class-Incremental Learning

FSCIL combines the strengths of FSL and CIL, enabling models to continually learn new classes using only a small number of labeled samples while retaining previously acquired knowledge (Zhang et al., 2025). This task was formally defined by, who established a benchmark protocol and highlighted the compounded challenges introduced by simultaneous data scarcity and class-incremental expansion.

Representative FSCIL methods address this challenge from multiple perspectives: TOPIC (Tao et al., 2020) introduces a topology-preserving mechanism (via neural gas) to stabilize the feature-space structure across incremental stages; CEC proposes continuously evolving classifiers to mitigate forgetting and maintain coherent decision boundaries (Zhang C. et al., 2021) Meta-learning-based frameworks (e.g., MetaFSCIL; Chi et al., 2022) and forward-compatible training (e.g., FACT; Zhou D. et al., 2022) enhance adaptability by improving cross-session generalization and by calibrating representations or classifiers for future updates.

These methods have achieved significant progress on widely used benchmark datasets, which are CUB-200-2011, miniImageNet, and CIFAR-100. However, most FSCIL baselines are still primarily benchmarked on general-purpose natural-image datasets under relatively controlled acquisition conditions. Such settings can differ substantially from scientific or ecological microscopy imagery, where fine-grained morphology, non-uniform illumination, imaging noise, background impurities, and skewed class distributions are common and can stress robustness and stability. The demonstration of the FSCIL workflow is shown in Figure 1.

### 2.4 Environmental microorganism recognition

Environmental microorganism recognition has progressed rapidly from classical pipelines based on hand-crafted descriptors (Ciranni et al., 2024) to modern end-to-end learning frameworks (Zhang et al., 2023). Recent advances increasingly leverage deep convolutional networks and vision transformers, often coupled with metric learning and attention mechanisms to better capture fine-grained morphology. To reduce reliance on exhaustive annotations, self-supervised and contrastive pretraining, semi-weakly supervised learning, and domain adaptation have become common strategies for extracting transferable visual representations from large collections of unlabeled or heterogeneously labeled microbial images (Bendidi et al., 2024). In parallel, data-centric solutions such as strong augmentation, synthetic sample generation, and uncertainty-aware sampling further improve robustness under class imbalance and imaging variability–conditions that are especially prevalent in environmental microbiology (Panaïotis et al., 2025).

Despite these methodological improvements, environmental monitoring imposes a fundamental operational constraint: models are typically trained in a static, closed-set manner and therefore can only recognize categories present in the training data, while real deployments often encounter samples from categories that were not included during training due to task diversity and incomplete coverage. Moreover, when such out-of-training categories are encountered, only a small number of labeled instances are usually available, making adaptation under sparse supervision essential. In contrast, many standard CIL settings implicitly assume that each incremental phase provides a non-trivial amount of training data, which can be unrealistic for environmental monitoring scenarios where newly encountered categories may be represented by only a handful of specimens. Conversely, FSL is designed for extreme data scarcity but does not, by itself, address the sequential acquisition of new categories and the need to preserve performance on previously learned classes (Wang et al., 2020).

This mismatch creates a critical gap for real-world environmental microorganism recognition: systems must simultaneously (i) learn novel microbial classes from very few images and (ii) retain prior knowledge over a long horizon without catastrophic forgetting (Zhou et al., 2024). FSCIL directly targets this dual requirement, making it particularly important for environmental applications where pronounced intra-class variability and subtle inter-class differences are common. Consequently, positioning FSCIL as a core paradigm–and establishing a dedicated FSCIL benchmark for environmental microbial imagery–is essential for aligning advanced AI recognition capabilities with the dynamic, long-term monitoring demands of environmental science.

## 3 Benchmark Construction

**Figure 3:**
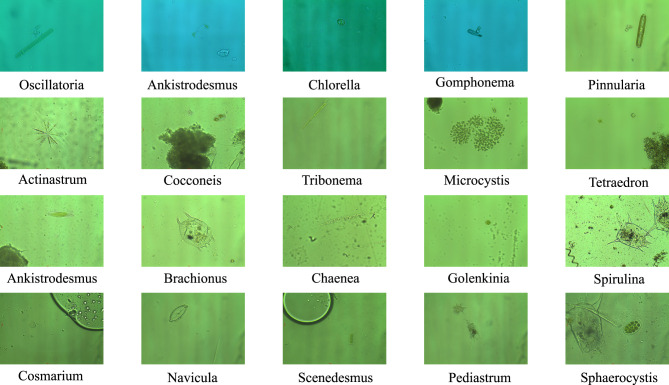
Some examples from the EMDS-7 dataset. Representative microscopic images of environmental microorganisms from the EMDS-7 dataset are illustrated. The dataset covers diverse microorganism categories with significant variations in morphology, scale, and texture. Meanwhile, several categories exhibit high inter-class similarity and complex visual patterns, which introduce fine-grained recognition challenges and increase the difficulty of FSCIL for environmental microorganism image recognition.

To establish a standardized, reproducible FSCIL evaluation platform for environmental microorganism image recognition, this study constructs a comprehensive FSCIL benchmark based on the EMDS-7 dataset. This section provides a detailed description of the data preprocessing pipeline, incremental task design, few-shot sampling strategy, and standardized evaluation protocols for assessing algorithm performance. The benchmark is designed to reflect practical ecological monitoring settings, where models trained on a fixed set of categories may encounter previously unseen categories with only a few labeled examples and must incorporate them without degrading performance on previously learned categories. Example images from the EMDS-7 dataset are shown in Figure 3.

### 3.1 Dataset preprocessing

All 41 categories are partitioned into a base session and multiple incremental sessions. The base session includes 20 categories, with 25 samples per category, for fully supervised representation learning to establish a robust feature space for subsequent increments. The remaining 21 categories are organized into seven incremental sessions, each introducing three novel categories under a 3-way 3-shot setting (Zhang et al., 2024), which mirrors the sparse, progressive inclusion of previously unseen categories encountered in practical microbial monitoring. The choice of the 3-shot incremental configuration follows the standard FSCIL protocol introduced by (Zhang et al., 2024), where each new class is represented by only three support samples to simulate extreme data scarcity during deployment. To reduce evaluation bias, the category grouping is constructed to maintain taxonomic diversity and comparable difficulty across sessions. The sampling ratio used in the base session follows the widely adopted 60%–40% train–test division described by Tao et al. (2020) for CIFAR-100 and miniImageNet, ensuring that EMDS-7 is aligned with canonical FSCIL dataset construction principles and remains directly comparable to prior work. Overall, this session design follows standard FSCIL protocols and enables controlled yet realistic assessment of how models adapt to new categories while preserving prior knowledge.

In this benchmark, EMDS-7 is converted into an image-level classification setting. Specifically, each original image is treated as one classification sample, without performing bounding-box cropping or object-level filtering. This design choice avoids introducing object-detection–dependent biases and ensures that all methods receive identical, unaltered visual evidence. All images are normalized and resized to a fixed resolution of 224 × 224 before being used for training and evaluation. This unified preprocessing avoids introducing design-dependent biases and ensures a standardized input format across all FSCIL methods. To ensure reproducibility, all processed images, session-wise class partitions, and fixed train/test split lists are included in the released benchmark resources.

### 3.2 Training and Evaluation Protocol

We follow a widely adopted and challenging FSCIL evaluation protocol to ensure rigor and comparability (Rebuffi et al., 2017). For fair comparison, all baselines use an ImageNet-pretrained ResNet-18 backbone. In the base stage, the model is trained on all images from the base categories for 100 epochs using SGD with an initial learning rate of 0.1 and standard learning rate decay. During incremental learning, we employ a frozen-backbone setting: backbone parameters remain fixed across sessions, and only the classifier is updated using the current session's 3-shot samples (Zhang C. et al., 2021). The base-stage training configuration (100 epochs with an initial learning rate of 0.1) follows widely adopted settings in existing FSCIL benchmarks and the original implementations of representative methods, and provides stable convergence on EMDS-7 without altering the relative performance ranking among methods. After each session, the model is evaluated on a cumulative test set containing all seen classes. We report *Average Accuracy* (AA), defined as the mean accuracy across all test sessions (Tao et al., 2020), and *Performance Drop* (PD), measured as the difference in accuracy between the base and final sessions, to quantify long-term stability and forgetting. This evaluation protocol corresponds to the Session-ID setting, where performance is assessed cumulatively after each incremental session, ensuring a consistent and transparent measurement of class-incremental behavior. All results are averaged over five random runs and summarized with performance evolution curves and tables.

We adopt the Session-ID evaluation protocol, where after each incremental sessions, the model is evaluated on a cumulative test set containing all classes observed up to that session. The Session-ID accuracy is defined as the top-1 classification accuracy on this cumulative set. Average Accuracy (AA) is computed as the mean of Session-ID accuracies across all sessions, and Performance Drop (PD) is defined as the difference between the base-session accuracy and the final-session accuracy. These metrics jointly quantify overall effectiveness and long-horizon stability under class-incremental learning.

## 4 Experimental results and analysis

**Figure 4:**
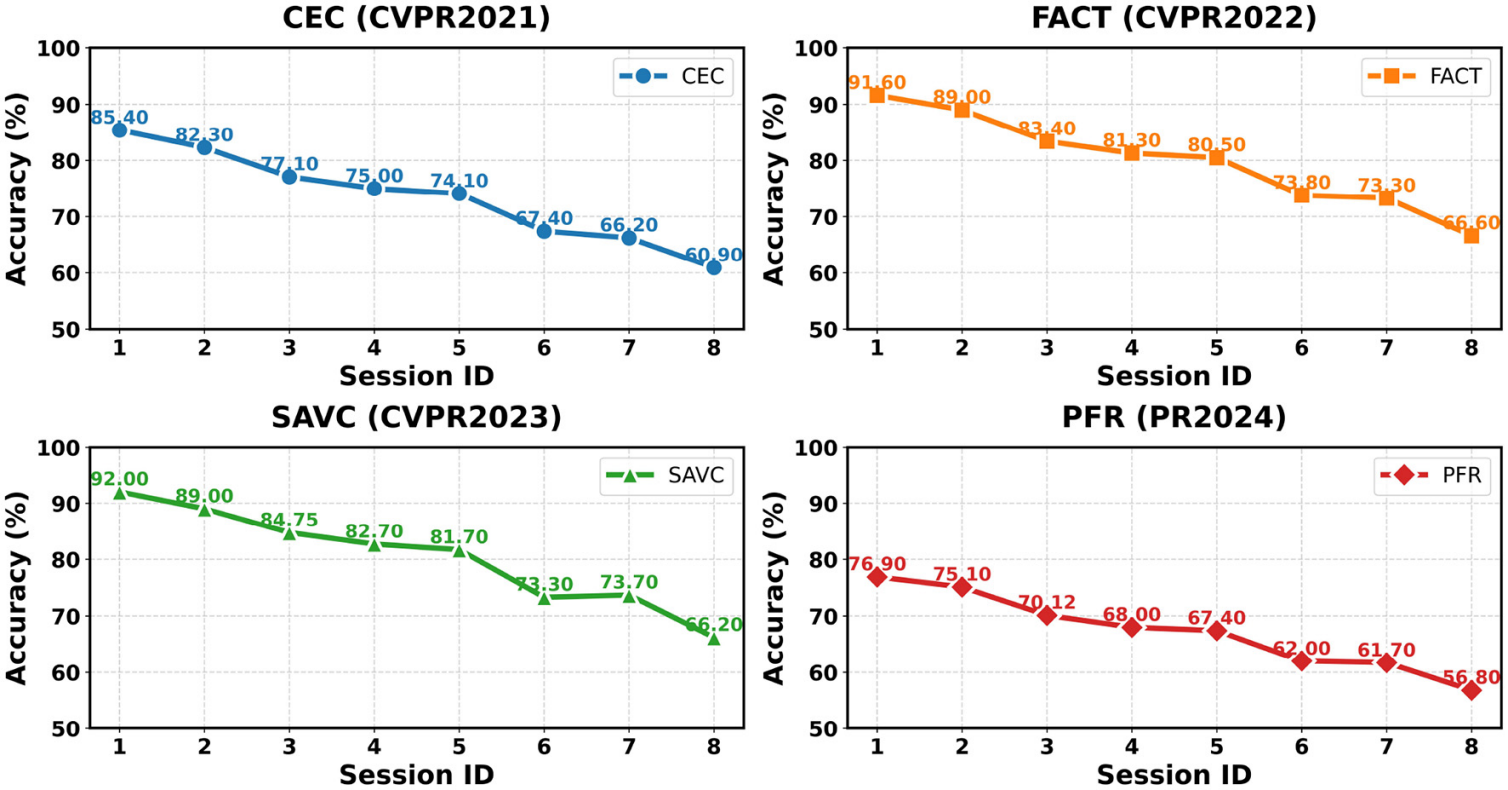
The comparison of the results of CEC, FACT, SAVC, and PPR on the EMDS-7 dataset.

**Figure 5:**
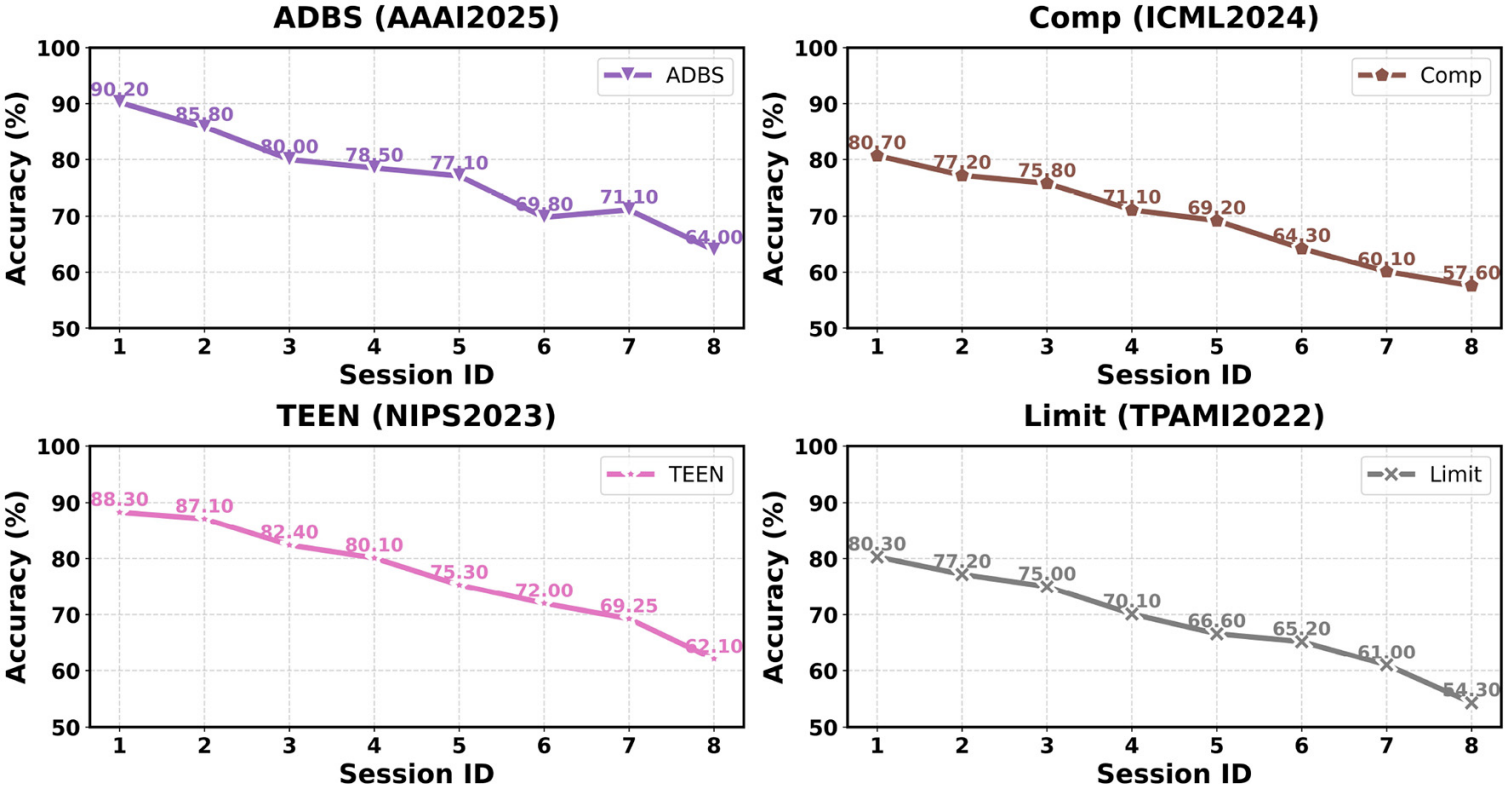
The comparison of the results of ADBS, Comp, TEEN, and Limit on the EMDS-7 dataset.

**Figure 6:**
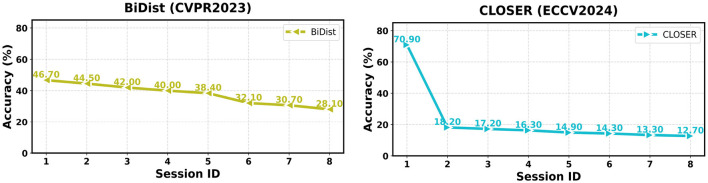
The comparison of the results of BiDist and CLOSER on the EMDS-7 dataset.

In this section, we compare representative FSCIL methods on the EMDS-7 dataset under the Session-ID evaluation protocol. We report both session-wise accuracies and aggregated metrics to characterize each method's overall effectiveness and long-horizon stability. As shown in Table 1, we provide session-wise accuracies (Sessions 0–7) together with AA and PD. Figures 4–6 visualize the corresponding accuracy curves. For a concise summary, Table 2 reports an overall ranking based on AA (higher is better) and PD (lower is better), along with each method's core mechanism.

### 4.1 Experimental setup

#### 4.1.1 Compared methods

We reproduce and compare 10 representative FSCIL methods spanning major design routes: classifier evolution (CEC), forward-compatible training (FACT), virtual class contrastive learning (SAVC), frequency-domain decomposition (PFR), adaptive decision boundary (ADBS), compositional learning (Comp), training-free calibration (TEEN), meta-task calibration (Limit), bilateral knowledge distillation (BiDist), and inter-class distance minimization (CLOSER). Following the visualization protocol in Figures 4–6, we group the accuracy curves into three panels for clearer trend inspection: (CEC, FACT, SAVC, PFR) in Figure 4, (ADBS, Comp, TEEN, and Limit) in Figure 5, and (BiDist, CLOSER) in Figure 6.

### 4.2 Quantitative results

#### 4.2.1 Overall comparison

As reported in Table 1, SAVC and FACT achieve the best overall *AA* on EMDS-7, reaching 80.4 and 79.9%, respectively. ADBS and TEEN follow next, with both methods attaining 77.1% *AA*. CEC shows mid-level performance with an *AA* of 73.5%, while Comp and Limit yield moderate *AA* values of 69.5 and 68.7%. Among the top and mid-performing methods, PFR exhibits the smallest *PD*, at 20.1%, but its *AA* remains lower at 67.2%, indicating stronger long-horizon stability but a limited separability ceiling. Compared with the above methods, BiDist operates at much lower accuracy, with an *AA* of 37.8% and a *PD* of 18.6%. CLOSER performs worst overall, with an *AA* of 22.2% and a substantially larger drop, where *PD* reaches 58.2%, suggesting markedly different failure patterns from the higher-performing group.

**Table 1:** Performance comparison of methods in the Session ID format.

**Method**	**Venue**	**Session ID**								**AA ↑**	**PD ↓**
		**0**	**1**	**2**	**3**	**4**	**5**	**6**	**7**		
CEC	CVPR2021	85.4	82.3	77.1	75.0	74.1	67.4	66.2	60.9	73.5	24.5
FACT	CVPR2022	91.6	89.0	83.4	81.3	80.5	73.8	73.3	66.6	79.9	25.0
SAVC	CVPR2023	92.9	89.0	84.7	82.7	81.7	73.3	73.7	66.2	80.4	26.7
PFR	PR2024	76.9	75.1	70.1	68.0	67.4	62.0	61.7	56.8	67.2	20.1
ADBS	AAAI2025	90.2	85.8	80.0	78.5	77.1	69.8	71.1	64.0	77.1	26.2
Comp	ICML2024	80.7	77.2	75.8	71.1	69.2	64.3	60.1	57.6	69.5	23.1
TEEN	NIPS2023	88.3	87.1	82.4	80.1	75.3	72.0	69.2	62.1	77.1	26.2
Limit	TPAMI2024	80.3	77.2	75.0	70.1	66.6	65.2	61.0	54.3	68.7	26.0
BiDist	CVPR2023	46.7	44.5	42.0	40.0	38.4	32.1	30.7	28.1	37.8	18.6
CLOSER	ECCV2024	70.9	18.2	17.2	16.3	14.9	14.3	13.3	12.7	22.2	58.2

**Table 2:** Performance ranking based on *AA* and *PD*. (*In* %).

**Rank**	**Method**	**Core mechanism**	** $A_{0}$ **	** $A_{final}$ **	**PD (↓)**	**AA (↑)**
1	SAVC	Virtual class contrastive learning	92.9	66.2	26.7	80.4
2	FACT	Forward compatible training	91.6	66.6	25.0	79.9
3	ADBS	Adaptive decision boundary	90.2	64.0	26.2	77.1
4	TEEN	Training-free calibration	88.3	62.1	26.2	77.1
5	CEC	Classifier graph evolution	85.4	60.9	24.5	73.5
6	Comp	Compositional learning	80.7	57.6	23.1	69.5
7	LIMIT	Meta-task calibration	80.3	54.3	26.0	68.7
8	PFR	Frequency domain decomposition	76.9	56.8	20.1	67.2
9	CLOSER	Inter-class distance minimization	70.9	12.7	58.1	22.2
10	BiDist	Bilateral knowledge distillation	46.7	28.1	37.8	18.6

#### 4.2.2 Session-wise trends

The accuracy curves in Figures 4–6 further clarify how different design choices affect long-horizon behavior. In Figure 4, SAVC and FACT start from very high base-session accuracy and maintain strong early-to-mid performance, whereas PFR exhibits a more gradual, near-linear decay, consistent with a stability-oriented design. In Figure 5, ADBS and TEEN remain competitive into later sessions with distinct stabilization patterns, while Comp and Limit show a more pronounced decline toward the final sessions. Figure 6 highlights the substantially lower accuracy regimes of BiDist and CLOSER. BiDist degrades smoothly across sessions, with accuracy decreasing from 46.7 to 28.1%. By contrast, CLOSER collapses immediately after the first incremental session, dropping from 70.9 to 18.2%, and then remains at a persistently low level thereafter.

### 4.3 Results analysis and discussion

#### 4.3.1 Accuracy-stability trade-off on EMDS-7

Table 1 and Figures 4–6 reveal a clear accuracy-stability trade-off on EMDS-7. This trade-off reflects a fundamental interaction between FSCIL method design principles and the intrinsic characteristics of environmental microorganism imagery, rather than isolated empirical variations across methods. Representation-oriented methods such as SAVC and FACT reach the highest accuracy ceiling, with *AA* values of 80.4 and 79.9%, but they also incur relatively large performance drops across sessions, where *PD* rises to 26.7 and 25.0%. In contrast, PFR shows a smaller drop, with *PD* measured at 20.1%, suggesting better long-horizon stability, yet its overall *AA* remains lower at 67.2%, reflecting limited separability in this fine-grained setting. From a methodological perspective, this pattern indicates that on EM imagery, higher *AA* primarily depends on strong base-session discriminability induced by representation learning mechanisms, whereas lower *PD* benefits more from stabilization strategies that explicitly constrain cumulative drift.

#### 4.3.2 Top performers: SAVC and FACT

SAVC achieves the highest base-session accuracy of 92.9% and remains strong through Sessions 1–4, where performance decreases from 89.0 to 81.7%. This trend indicates that contrastive learning in virtual classes substantially improves fine-grained separability. From a method-design standpoint, SAVC emphasizes aggressive feature discrimination during the base session, which is particularly effective for EMDS-7 where subtle morphological cues dominate class differentiation. However, SAVC exhibits a pronounced drop between Sessions 4 and 5, from 81.7 to 73.3%, and converges to 66.2% in the final session, consistent with the sharper late-stage decline shown in Figure 4. Under dense near-neighbor class structures and extremely low-shot increments, such highly discriminative feature geometry can accumulate boundary crowding and prototype drift, leading to increasingly severe old–new conflicts in later sessions.

FACT starts from a similarly high base accuracy and reaches 91.6%, then degrades more smoothly in early-to-mid sessions, with accuracy decreasing from 89.0 to 80.5%. This aligns with its forward-compatible design that delays interference. FACT explicitly attempts to reserve decision space for future classes, which moderates early interference compared with more rigid representation shaping. Nevertheless, FACT still drops to 66.6% at the final session, implying that space reservation alone cannot fully resolve decision boundary overlap induced by tightly clustered fine-grained classes under long incremental chains.

#### 4.3.3 Second tier: ADBS and TEEN

ADBS and TEEN reach the same *AA* value of 77.1% but exhibit different stabilization patterns in Figure 5. ADBS produces more controllable curves and shows a mild rebound at Session 6, rising to 71.1% from 69.8% at Session 5. This behavior is consistent with adaptive boundary adjustment that mitigates old-class dominance and reduces old-new conflicts. From a methodological perspective, ADBS dynamically reshapes decision boundaries, allowing limited plasticity while preventing excessive drift, which is advantageous in long-horizon incremental settings. TEEN maintains strong early performance, reaching 87.1% at Session 1, suggesting that training-free calibration can alleviate the initial attraction of novel classes to old ones. However, TEEN continues to decline along the long incremental chain and drops to 62.1% at Session 7, suggesting that prototype-level post-processing alone may accumulate calibration errors in the highly ambiguous EMDS-7 space.

#### 4.3.4 Stable but ceiling-limited methods

CEC achieves an *AA* of 73.5% and performs reasonably well in early sessions, but its accuracy drops to 60.9% in the final session. This behavior is consistent with error accumulation in classifier evolution or relationship propagation under dense near-neighbor structures. Comp and Limit operate in a similar mid-range regime, yielding *AA* values of 69.5 and 68.7%, respectively, and neither shows a clear advantage in terms of ceiling or stability on EMDS-7. Comp demonstrates some robustness, with *PD* reaching 23.1%, but its overall separability remains limited, whereas Limit does not outperform other methods on either *AA* or *PD*. PFR, although achieving a lower *AA*, exhibits improved stability, with *PD* measured at 20.1%. From a method-design perspective, frequency-domain decomposition suppresses certain forms of cumulative drift by stabilizing texture-dominant cues in EM imagery, but this comes at the cost of reduced spatial discriminability, limiting the achievable accuracy ceiling.

#### 4.3.5 Low-ceiling vs. failure-like behaviors

BiDist and CLOSER exhibit markedly different non-top-performing behaviors (Figure 6). BiDist shows the smallest *PD* in our comparison, with a drop of 18.6%, while its overall *AA* remains substantially lower at 37.8%, indicating a relatively stable yet low-ceiling regime. This pattern is consistent with a distillation-dominated optimization that preserves earlier decision structure but constrains the model's capacity to carve out discriminative regions for newly introduced classes under the 3-way 3-shot setting.

More generally, distillation-dominated methods emphasize stability by preserving historical decision boundaries, but this inductive bias inevitably limits plasticity when discriminative cues between novel classes are subtle and data is extremely scarce. As a result, such methods tend to trade accuracy ceiling for improved long-term retention on EMDS-7.

In contrast, CLOSER undergoes a sharp degradation immediately after the first incremental session, falling from 70.9 to 18.2%, and remains at a very low level thereafter; consequently, it yields a large *PD* of 58.2% together with a low *AA* of 22.2%. Such a trajectory suggests that inter-class distance minimization may become incompatible with EMDS-7's compact, fine-grained class geometry when incremental evidence is extremely scarce. From a methodological perspective, CLOSER explicitly enforces geometric constraints on feature space by minimizing inter-class distances, implicitly assuming reliable and well-separated class prototypes.

However, EMDS-7 exhibits subtle inter-class differences and substantial intra-class variability, leading to highly compact feature distributions across categories. Under the extremely low-shot (3-shot) incremental setting, class prototypes estimated from only a few samples are subject to high uncertainty. When strong geometric constraints are imposed under such conditions, prototype instability can be amplified, resulting in severe decision-boundary overlap and rapid performance collapse.

Beyond these two methods, similar method–data interactions consistently emerge across different FSCIL method families. Representation-driven approaches (e.g., SAVC and FACT) favor discriminability and achieve higher accuracy ceilings, whereas stabilization-oriented methods (e.g., ADBS, TEEN, and PFR) prioritize controlling drift and achieve improved long-horizon stability. Constraint-heavy designs, including aggressive distillation or geometric shaping, are particularly fragile under extreme data scarcity. Since all methods are reproduced using public implementations under a unified setup, these observations should be interpreted primarily as empirical signals of method-domain compatibility rather than definitive general conclusions.

#### 4.3.6 Summary

Overall, EMDS-7 highlights that achieving high *AA* relies on strong fine-grained discriminability in representation learning, such as SAVC and FACT, while achieving lower *PD* benefits more from lightweight stabilization and calibration, such as ADBS, TEEN, and PFR. From a method-design perspective, this indicates that balancing representation quality and controlled plasticity is critical for FSCIL in environmental microorganism recognition. In near-neighbor classes, with 3-shot increments, and in long-horizon sessions, overly aggressive geometric shaping or distillation constraints may introduce instability and lead to substantial degradation.

## 5 Conclusions and prospects

In this work, EMDS-7 is introduced to evaluate 10 representative methods under a unified Session-ID protocol systematically, which is the first FSCIL benchmark for environmental microbiology. The results show that environmental microbial images pose distinct challenges, with high intra-class diversity, subtle inter-class differences, and noisy backgrounds, which lead to clear method-dependent behaviors that differ from those on natural-image benchmarks.

A key takeaway is that strong performance on generic FSCIL datasets does not necessarily transfer to EMDS-7; rather, the effectiveness of a method depends on how well its underlying mechanism matches the dataset's compact, fine-grained class geometry. In our experiments, representation-driven approaches such as SAVC and FACT achieve the best overall performance, while calibration-based stabilizers (e.g., ADBS and TEEN) remain highly competitive by mitigating old–new conflicts over long session chains. Frequency-domain stabilization methods (e.g., PFR) improve long-term stability but are constrained by a lower separability ceiling, thereby highlighting an explicit accuracy–stability trade-off. Frequency-domain stabilization (e.g., PFR) improves stability but is constrained by a lower separability ceiling, highlighting an accuracy–stability trade-off.

Overall, EMDS-7 provides a solid experimental foundation and points to promising directio ns, including the need for task-specific FSCIL adaptations such as incorporating domain priors and improving stability–plasticity control in fine-grained, data-scarce settings.

## Data Availability

The original contributions presented in the study are included in the article/Supplementary material, further inquiries can be directed to the corresponding author.
