# Supplementary figures and images for: EMDS-7-FSCIL: a benchmark for Few-Shot Class-Incremental Learning in environmental microorganism recognition
Source: Front Microbiol. 2026 Feb 10;17:1770528. doi: 10.3389/fmicb.2026.1770528 (PMC12929393; doi:10.3389/fmicb.2026.1770528)

### BiDist (CVPR2023)

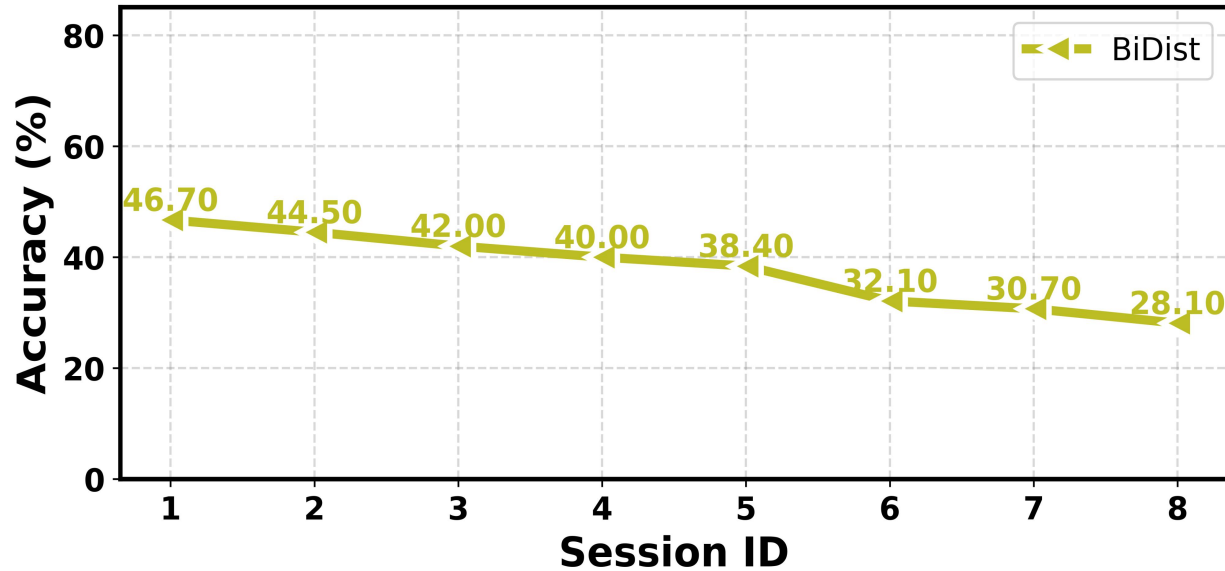

### CLOSER (ECCV2024)

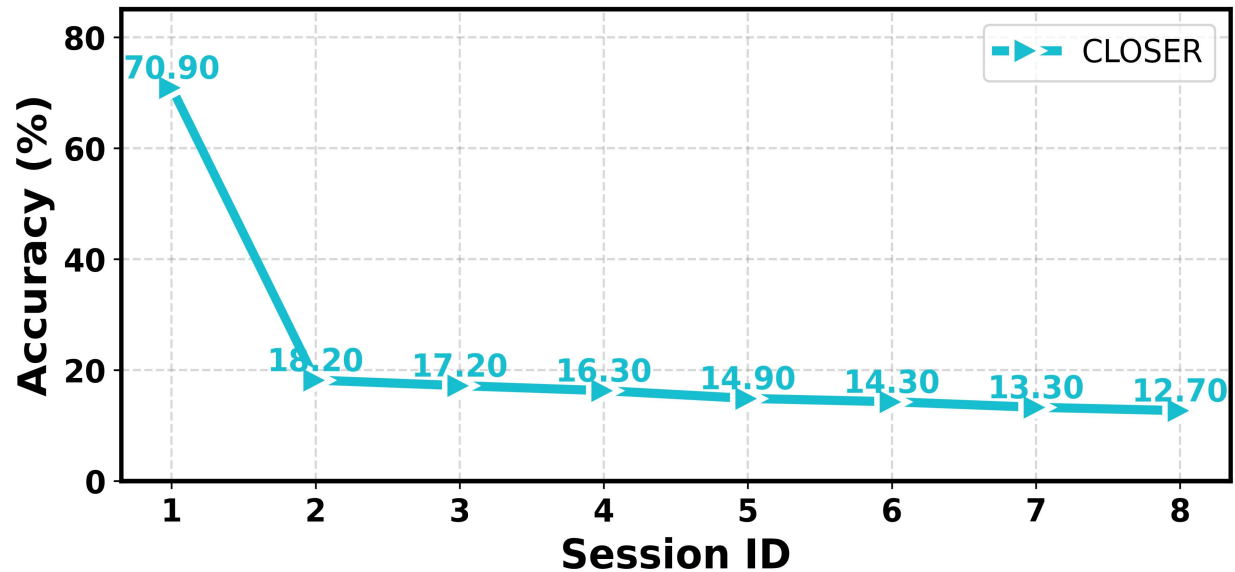

Supplement: Supplementary file 1 [file Data_Sheet_1.pdf]

**ADBS (AAAI2025)**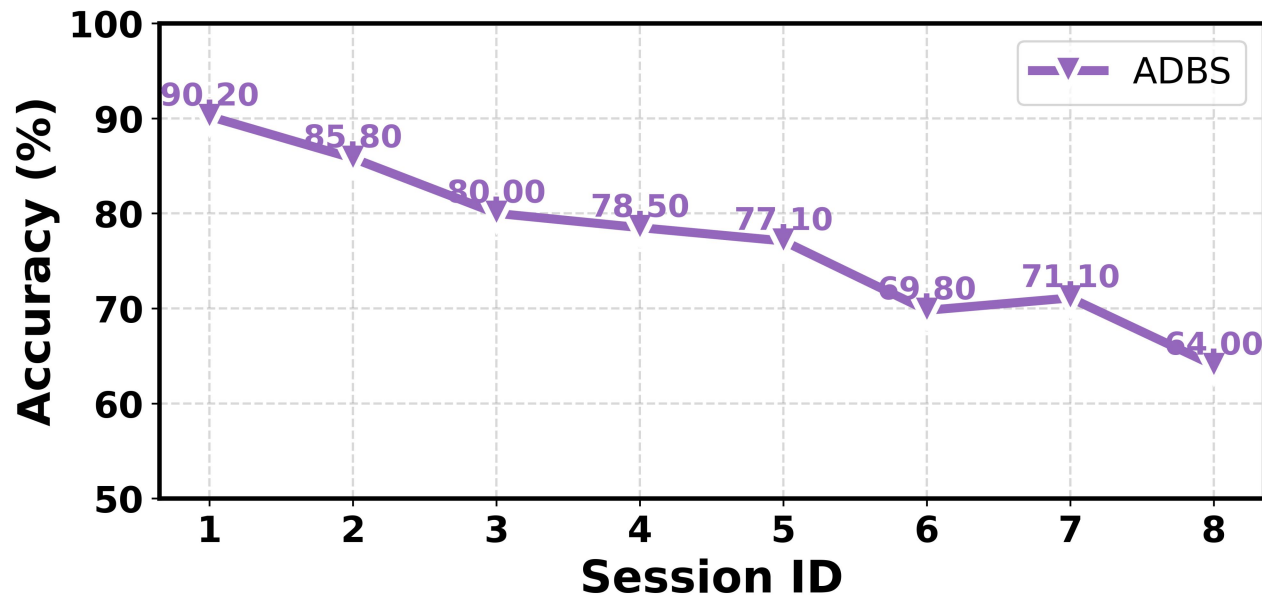**Comp (ICML2024)**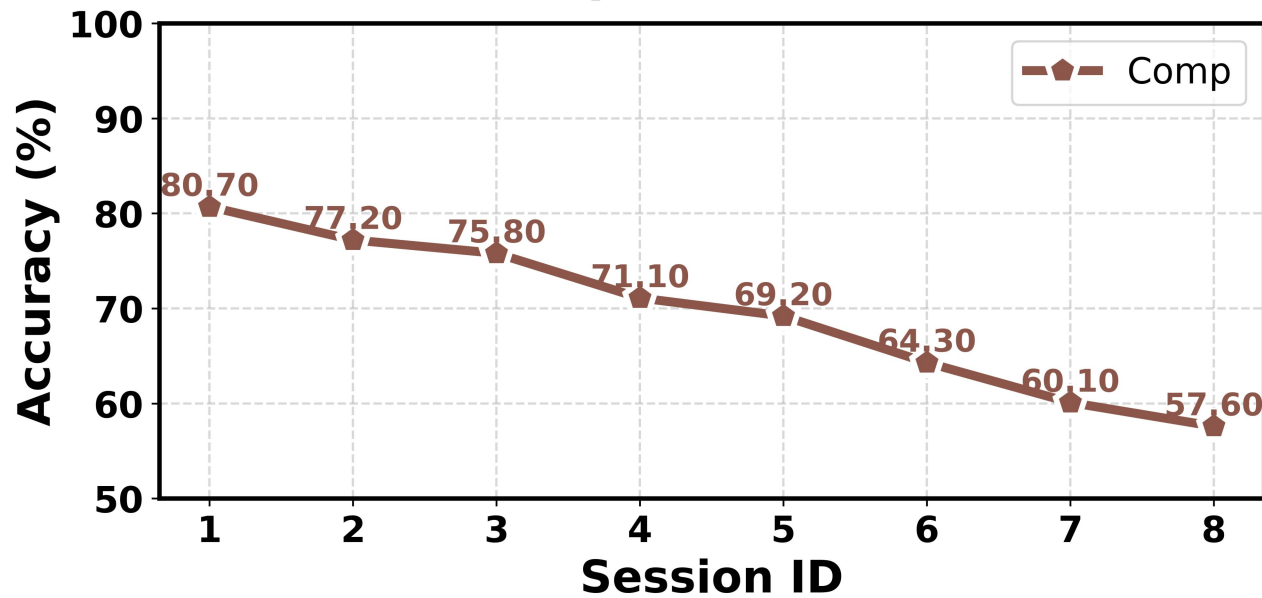**TEEN (NIPS2023)**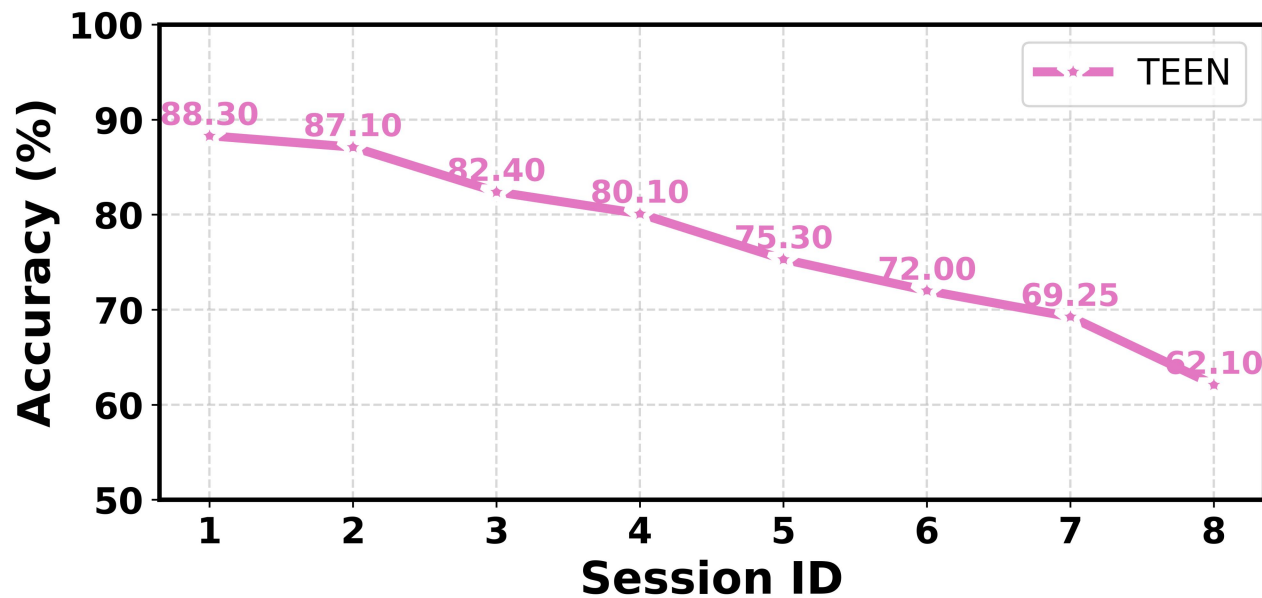**Limit (TPAMI2022)**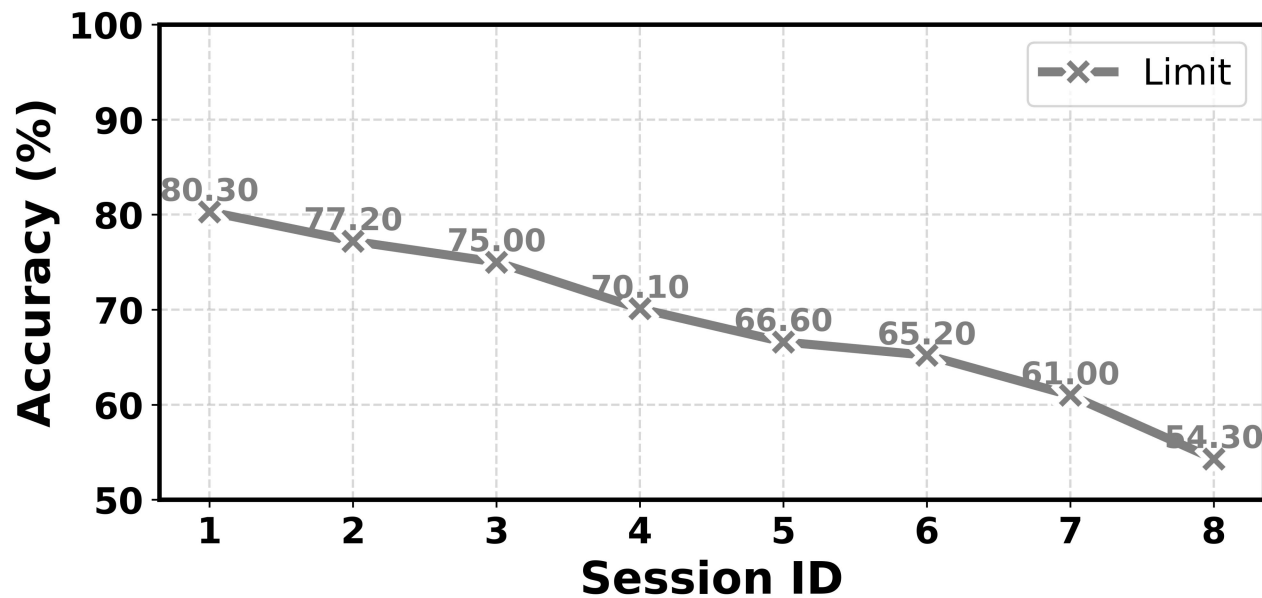

Supplement: Supplementary file 2 [file Data_Sheet_2.pdf]

**CEC (CVPR2021)**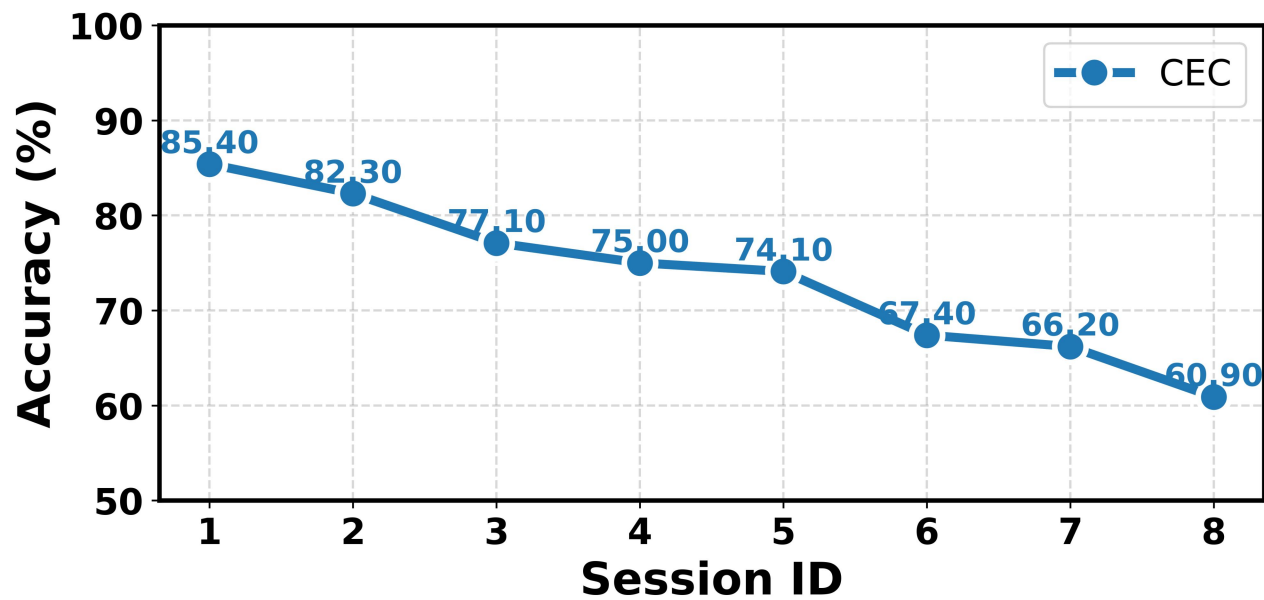**FACT (CVPR2022)**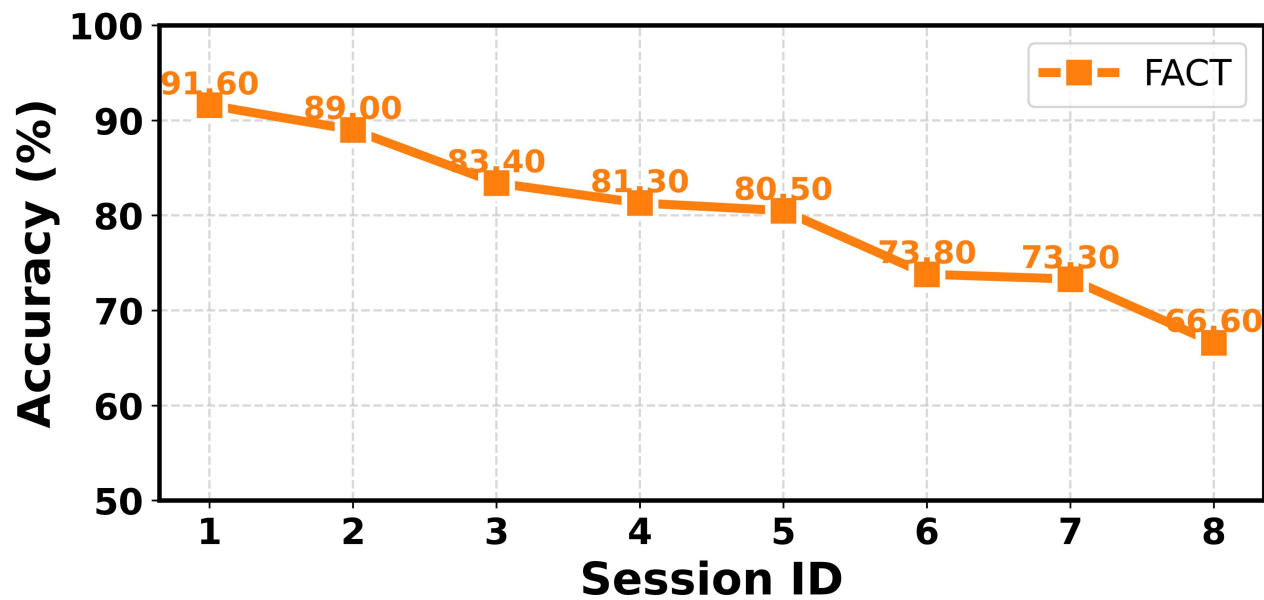**SAVC (CVPR2023)**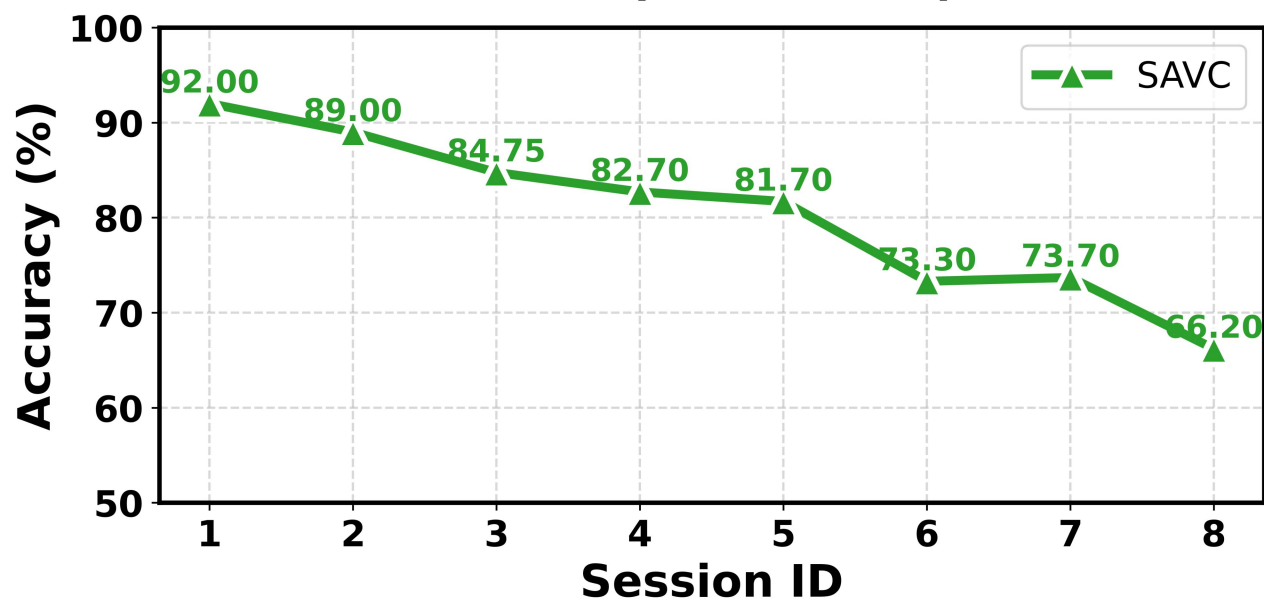**PFR (PR2024)**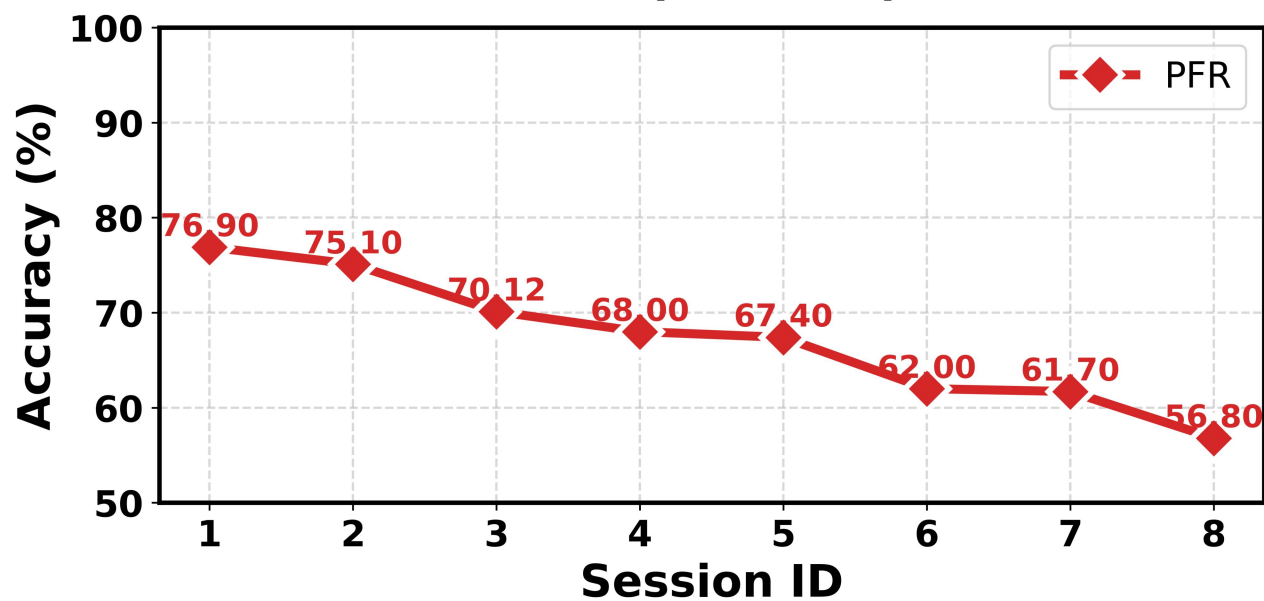

Supplement: Supplementary file 3 [file Data_Sheet_3.pdf]

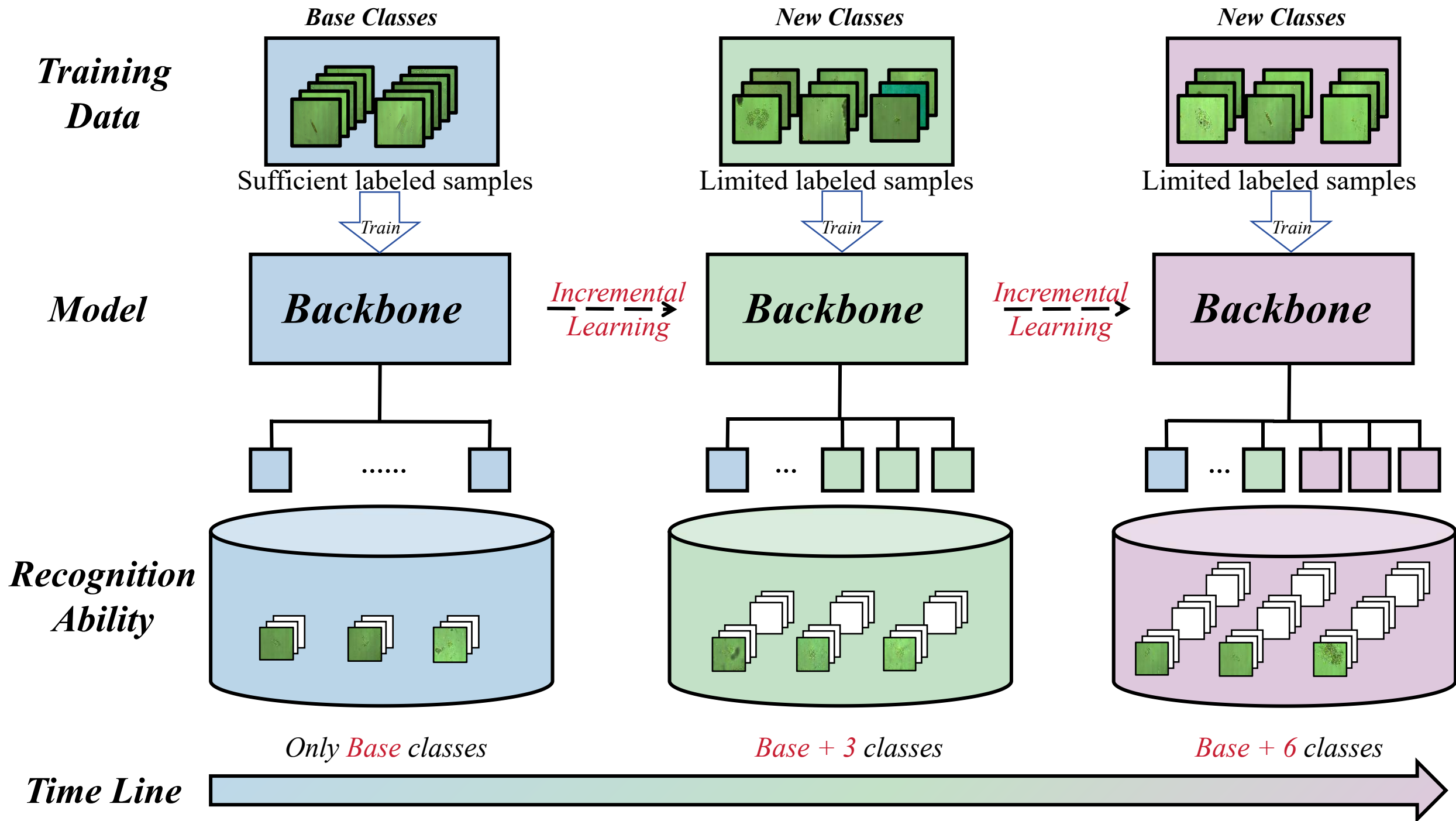

Supplement: Supplementary file 6 [file Data_Sheet_6.pdf]
