# Supplementary material for: EMDS-7-FSCIL: a benchmark for Few-Shot Class-Incremental Learning in environmental microorganism recognition
Source: Front Microbiol. 2026 Feb 10;17:1770528. doi: 10.3389/fmicb.2026.1770528 (PMC12929393; doi:10.3389/fmicb.2026.1770528)
Supplement: Supplementary file 4 [file Data_Sheet_4.pdf]

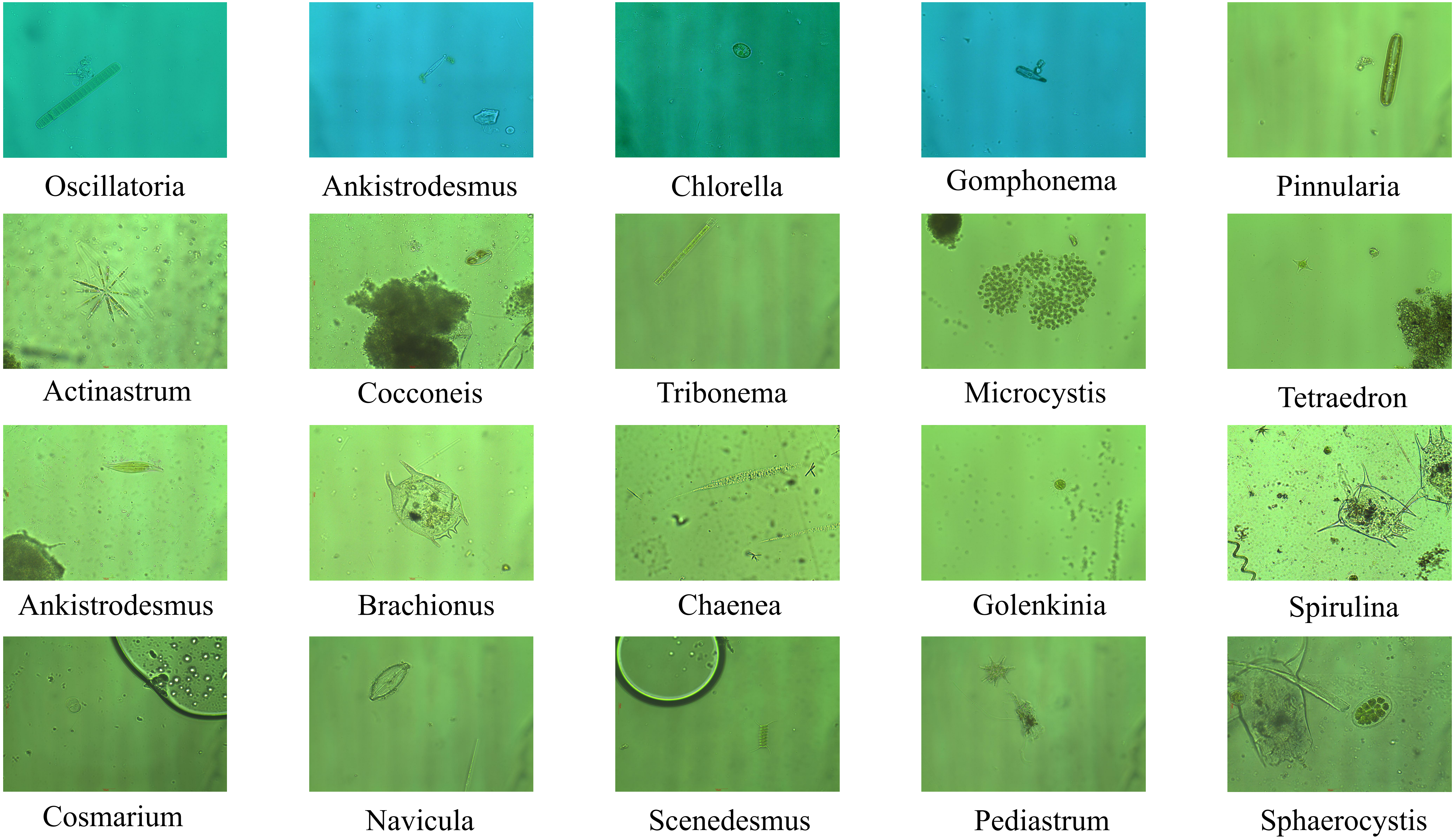

Oscillatoria
Ankistrodesmus
Chlorella
Gomphonema
Pinnularia
Actinastrum
Cocconeis
Tribonema
Microcystis
Tetraedron
Ankistrodesmus
Brachionus
Chaenea
Golenkinia
Spirulina
Cosmarium
Navicula
Scenedesmus
Pediastrum
Sphaerocystis
