# Supplementary material for: EMDS-7-FSCIL: a benchmark for Few-Shot Class-Incremental Learning in environmental microorganism recognition
Source: Front Microbiol. 2026 Feb 10;17:1770528. doi: 10.3389/fmicb.2026.1770528 (PMC12929393; doi:10.3389/fmicb.2026.1770528)
Supplement: Supplementary file 5 [file Data_Sheet_5.pdf]

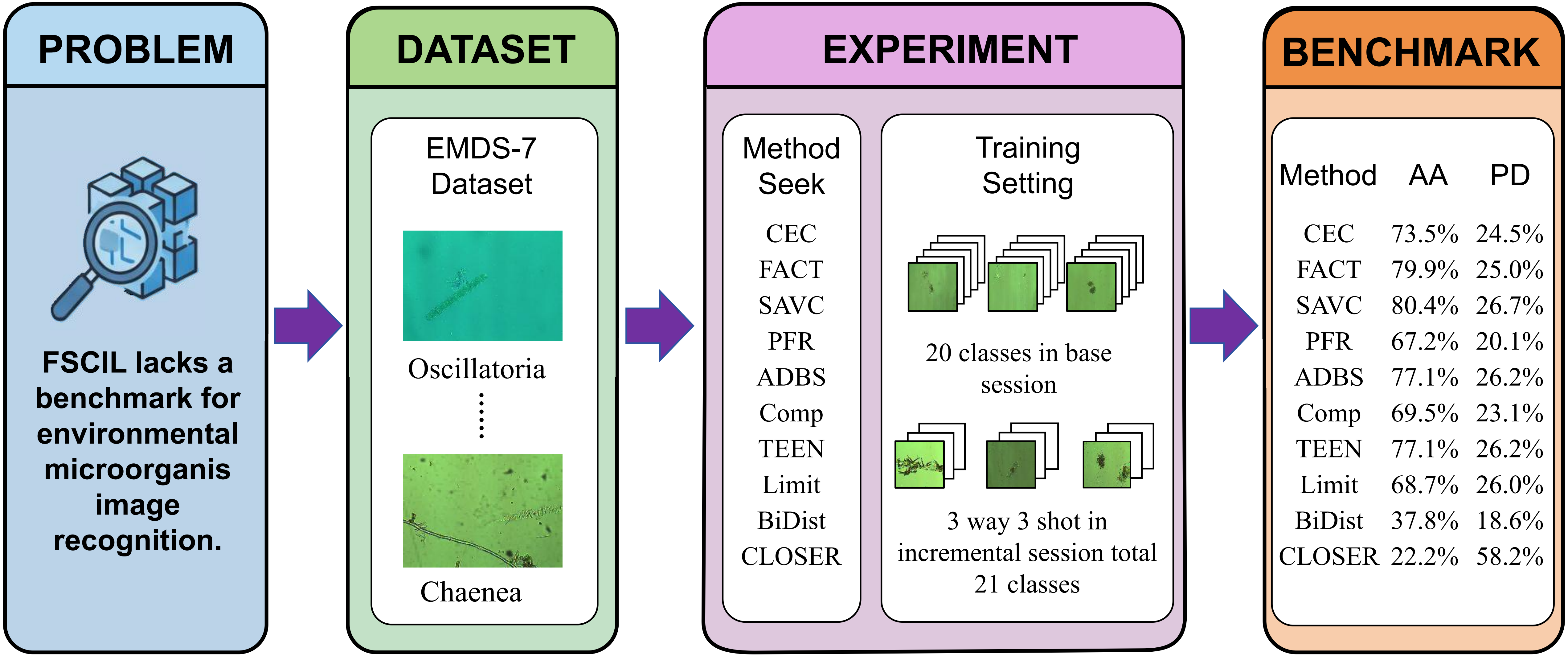
PROBLEM
FSCIL lacks a benchmark for environmental microorganis image recognition.
DATASET
EMDS-7 Dataset
Oscillatoria
Chaenea
EXPERIMENT
Method Seek
CEC
FACT
SAVC
PFR
ADBS
Comp
TEEN
Limit
BiDist
CLOSER
Training Setting
20 classes in base session
3 way 3 shot in incremental session total 21 classes
BENCHMARK
Method AA PD
CEC 73.5% 24.5%
FACT 79.9% 25.0%
SAVC 80.4% 26.7%
PFR 67.2% 20.1%
ADBS 77.1% 26.2%
Comp 69.5% 23.1%
TEEN 77.1% 26.2%
Limit 68.7% 26.0%
BiDist 37.8% 18.6%
CLOSER 22.2% 58.2%
